# CD19 directed CAR T cell therapy in diffuse large B-cell lymphoma

**DOI:** 10.18632/oncotarget.25688

**Published:** 2018-07-06

**Authors:** Alfonso Quintás-Cardama

**Affiliations:** Chief Medical Officer, TCR^2^ Therapeutics, Cambridge, MA, USA

**Keywords:** CAR T cell, lymphoma, axi-cel, tisagenlecleucel, liso-cel

Diffuse large B-cell lymphoma (DLBCL) represents 30-40% of all newly diagnosed cases of non-Hodgkin lymphoma (NHL). Chemoimmunotherapy with R-CHOP is curative in 50-60% of cases but most patients failing frontline therapy will perish to their disease, including those eligible for autologous stem cell transplantation (SCT) [1]. Chimeric antigen receptors (CARs) are synthetic protein constructs that contain an extracellular tumor antigen binding domain and intracellular activating elements, including a costimulatory domain such as 4-1BB or CD28 [2]. The choice of costimulatory domain impacts persistence (months for CD28 vs years for 4-1BB) and expansion (favored by CD28) *in vivo* [2]. CD19-targeted CAR T cells have proven effective in relapsed/refractory DLBCL. Two CARs, axicabtagene ciloleucel (axi-cel, YescartaTM, Gilead) and tisagenlecleucel (KymriahTM, Novartis) are approved in that setting and a third one, lisocabtagene maraleucel (liso-cel, Celgene), has generated promising preliminary data. Despite differences in costimulatory molecules, dosing, lymphodepletion, bridging therapy, patient population, and length of follow-up, multicenter trials have reported robust efficacy with all 3 CARs (Table 1).

**Table 1:** Multicenter clinical trials with CAR T cell therapies in relapsed/refractory aggressive NHL

	ZUMA-1	JULIET	TRANSCEND NHL-001	
Trial design & footprint	U.S & Israel (22 sites)	Global (27 sites, 10 countries)	U.S. (14 sites)	
Agent	Axi-cel	Tisagenlecleucel	Liso-cel	
Co-stimulation	CD28	4-1BB	4-1BB	
Bridging Therapy	Not allowed	Allowed	Allowed	
Lymphodepletion	Flu/Cy	Flu/Cy or Bendamustine	Flu/Cy	
CAR T cell dose	2×10^6^/kg	Median 3.1×10^8^ (range, 0.1-6)	DL1: 5×10^7^ DL2: 1×10^8^	
No. Patients	108*	81*	CORE (*n* = 67)	FULL (*n* = 91)
Indication	Refractory DLBCL, tFL, PBMCL	Relapsed or refractory DLBCL or tFL	r/r DLBCL NOS or tFL, 2-/3-hit NHL	CORE + tCLL/MZL, PMBCL, or FL3B
Best ORR (%)	82	53	74	
Best CR (%)	58	40	52	
3-mo ORR (%)	39	38	65	74
3-mo CR (%)	33	32	54	52
Ongoing CR (%)	40	30	42	53
Median DOR (months)	11.1	NR	9.2	44
Median OS (months)	NR	NR	NR	
Grade 3+ CRS (%)	13	23	1	1
Grade 3+ CRES (%)	28	12	15	12
Tocilizumab usage (%)	43	15	12	
CAR T related death (%)	3	0	0	

Abbreviations: CIT: chemo-immunotherapy; Flu: fludarabine; Cy: cyclophosphamide; No.: number; DLBCL: diffuse large B-cell lymphoma; t: transformed; FL: follicular lymphoma: CLL: chronic lymphocytic leukemia; MZL: marginal zone lymphoma; NOS: not otherwise specified; PMBCL: primary mediastinal B-cell lymphoma; ORR: overall response rate; CR: complete response; DOR: duration of response; OS: overall survival; CRS: cytokine release syndrome; CRES: CAR T cell-related encephalopathy syndrome; NR: not reported: NA: not applicable. *Evaluable patients.

In the pivotal ZUMA-1 trial, axi-cel was administered to 108 patients with refractory aggressive NHL [3]. T-cell products were manufactured with a 99% success rate and infused after a median of 17 days post-leukapheresis. Hospitalization was required for at least 7 days post-infusion for toxicity monitoring. The best ORR was 82% and the best CR was 58%. After a median follow-up of 15.4 months, 42% of patients continue to respond (40% in CR) and the OS rate at 18 months was 52% [3]. CRS occurred in 93% of patients (grade ≥3 in 13%) whereas neurotoxicity occurred in 64% of patients (grade ≥3 in 28%) [3]. Three axi-cel related deaths, including 2 due to severe CRS, were reported [3].

In the pivotal JULIET study, 99 patients with relapsed/refractory DLBCL received tisagenlecleucel [4]. Drop-out rates were high as tisagenlecleucel could only be given to 67% of enrolled patients. The best ORR and CR rates were 53% and CR 40%, respectively. The 6-month RFS was 74% and the median OS was not reached. CRS occurred in 58% of patients (grade 3/4 in 23%) and grade 3/4 neurotoxicity occurred in 12% of patients, but neither complication was fatal [4].

The TRANSCEND NHL-001 trial tested liso-cel at a fixed 1:1 CD4:CD8 ratio at two dose levels (5×107 and 1×108 cells) [5]. In the FULL dataset (*n* = 91), the best ORR was 74% and best CR rate was 52%. In the CORE dataset (*n* = 67), including only high-grade B-cell lymphoma (double/triple hit), DLBCL *not otherwise specified* either *de novo* or transformed from follicular lymphoma, the best ORR and CR rates were 80% and 55%. Grade 3/4 CRS and neurotoxicity rates were only 1% and 15%, respectively but 60% of patients had none of those toxicities, thus supporting the investigation of outpatient administration [6].

CD19-directed CARs improve upon salvage therapy for patients with DLBCL who have failed at least 2 lines of therapy [1]. The SCHOLAR-1 study reported an ORR of 26% and CR rate of 7% with standard salvage therapy in patients with refractory aggressive NHL [1]. Liso-cel appears to render a higher CR rate than axi-cel or tisagenlecleucel but longer follow-up in a pivotal trial is warranted to confirm these findings. Tisagenlecleucel and axi-cel are only available through Risk Evaluation and Mitigation Strategy (REMS) programs as severe CRS and/ or neurotoxicity afflict a significant number of patients, particularly those with high baseline tumor burden and/or levels of inflammation markers. The latter may provide a means to better select patients to minimize on-target/off-tumor toxicity. While axi-cel has been the first CAR T cell approved in DLBCL, this competitive advantage may be offset by the approval of safer options. Tisagenlecleucel is associated with a higher risk of severe CRS but lower risk of severe neurotoxicity and lesser tocilizumab usage than axi-cel. However, tisagenlecleucel has been associated with high drop-out rates, partly due to manufacturing inefficiencies leading to prolonged *vein-to-vein* times. Liso-cel appears to exhibit the safest toxicity profile of all available CD19-directed CARs (severe CRS rate 1%), and the potential for outpatient administration. If these preliminary results are confirmed in an ongoing pivotal trial and its *vein-to-vein* time is similar to that of axi-cel, it might become the *best-in-class* CAR product for DLBCL.

These studies have demonstrated the feasibility of manufacturing personalized cell therapies at a centralized facility and its delivery at a global scale. Nevertheless, only a few hundred patients with DLBCL have received CAR T cell therapy thus far, and all of them in the context of single-arm, uncontrolled studies with limited follow-up. The latter prevents making conclusions regarding the potential curative potential of these therapies, their long-term toxicity, and their activity compared to standard chemoimmunotherapy or autologous SCT. These are key considerations given the $373,000 price tag attached to both tisagenlecleucel and axi-cel. Since 7,500 patients with relapsed/refractory DLBCL are eligible for CAR T cell therapy, the total expenditure in the U.S. alone would exceed $3B [7]. Improvements in T cell leading to improved efficacy, lower CRS/neurotoxicity rates and severity that enable outpatient administration, a better understanding of the pathophysiology of those toxicities, and improved cheaper manufacturing processes represent the immediate challenges to improve current CAR T cell therapies.
